# Deriving Pulmonary Ventilation Images From Clinical 4D-CBCT Using a Deep Learning-Based Model

**DOI:** 10.3389/fonc.2022.889266

**Published:** 2022-05-02

**Authors:** Zhiqiang Liu, Yuan Tian, Junjie Miao, Kuo Men, Wenqing Wang, Xin Wang, Tao Zhang, Nan Bi, Jianrong Dai

**Affiliations:** National Cancer Center/National Clinical Research Center for Cancer/Cancer Hospital Chinese Academy of Medical Sciences and Peking Union Medical College, Department of Radiation Oncology, Beijing, China

**Keywords:** 4D-CBCT, pulmonary ventilation imaging, functional imaging, deep learning, image-guided radiotherapy

## Abstract

**Purpose:**

The current algorithms for measuring ventilation images from 4D cone-beam computed tomography (CBCT) are affected by the accuracy of deformable image registration (DIR). This study proposes a new deep learning (DL) method that does not rely on DIR to derive ventilation images from 4D-CBCT (CBCT-VI), which was validated with the gold-standard single-photon emission-computed tomography ventilation image (SPECT-VI).

**Materials and Methods:**

This study consists of 4D-CBCT and 99mTc-Technegas SPECT/CT scans of 28 esophagus or lung cancer patients. The scans were rigidly registered for each patient. Using these data, CBCT-VI was derived using a deep learning-based model. Two types of model input data are studied, namely, (a) 10 phases of 4D-CBCT and (b) two phases of peak-exhalation and peak-inhalation of 4D-CBCT. A sevenfold cross-validation was applied to train and evaluate the model. The DIR-dependent methods (density-change-based and Jacobian-based methods) were used to measure the CBCT-VIs for comparison. The correlation was calculated between each CBCT-VI and SPECT-VI using voxel-wise Spearman’s correlation. The ventilation images were divided into high, medium, and low functional lung regions. The similarity of different functional lung regions between SPECT-VI and each CBCT-VI was evaluated using the dice similarity coefficient (DSC). One-factor ANONA model was used for statistical analysis of the averaged DSC for the different methods of generating ventilation images.

**Results:**

The correlation values were 0.02 ± 0.10, 0.02 ± 0.09, and 0.65 ± 0.13/0.65 ± 0.15, and the averaged DSC values were 0.34 ± 0.04, 0.34 ± 0.03, and 0.59 ± 0.08/0.58 ± 0.09 for the density change, Jacobian, and deep learning methods, respectively. The strongest correlation and the highest similarity with SPECT-VI were observed for the deep learning method compared to the density change and Jacobian methods.

**Conclusion:**

The results showed that the deep learning method improved the accuracy of correlation and similarity significantly, and the derived CBCT-VIs have the potential to monitor the lung function dynamic changes during radiotherapy.

## Introduction

The side effects of damage to normal lung tissue limit the delivered dose for thoracic cancer patients during radiotherapy treatment and may thus hamper tumor control. It is necessary to assess lung function during radiotherapy. Ventilation imaging can quantify vital lung function regions and be useful for functional avoidance in thoracic cancer radiotherapy treatment planning (1, 2), or adaptive ventilation-guided radiotherapy on measuring how the lung ventilation changes during treatment (3).

There are several typical standard techniques, including single-photon emission computed tomography (4) (SPECT), positron emission tomography (5) (PET), and magnetic resonance imaging (6) (MRI), for acquiring the spatial ventilation images, but those imaging techniques are not commonly applied during radiation therapy of thoracic cancer in clinical practice. Ventilation images derived from four-dimensional computed tomography (4D-CT) scans are already available (7, 8), in many institutions and routinely acquired for thoracic cancer patients before radiotherapy, which can be used for functional avoidance radiotherapy treatment planning. The four-dimensional cone-beam computed tomography (4D-CBCT) imaging is already available for many institutions, and this can be taken as part of the daily patient position verification and motion management during radiotherapy. Ventilation images derived from 4D-CBCT (CBCT-VI) have the potential to provide details on lung function changes during radiotherapy. These kinds of images have a higher resolution and no extra monetary or dosimetric cost to the patient compared with typical standard techniques. Earlier studies have investigated variations over time in 4D-CBCT-based ventilation measures (9). It has also been shown that ventilation derived from high-quality 4D-CBCT scans has high correlations with ventilation derived from 4D-CT (10, 11).

However, there is no validation of CBCT-VI with a clinical gold-standard ventilation image. Furthermore, the current algorithms on deriving a ventilation image are highly dependent on deformable image registration (DIR) (8, 12, 13). The reduced image quality of 4D-CBCT compared to 4D-CT further impacts the precision of the DIR. Inaccuracies in the DIR can lead directly to errors in deriving a ventilation image. This study proposes a DIR-independent deep learning model for deriving ventilation images from 4D-CBCT, makes a clinical validation against the gold-standard SPECT ventilation image (SPECT-VI), and also investigates whether the accuracy could be improved compared to DIR-dependent methods.

## Materials and Methods

### Data Collection

This study consists of 4D-CBCT and SPECT/CT scans of 28 patients with esophagus or lung cancer who underwent thoracic radiotherapy in our hospital between 2015 and 2018. All patient images were acquired with approval by the Clinical Research Committee and the Ethics Committee at the Cancer Hospital, Chinese Academy of Medical Sciences. The 4D-CBCT images were acquired on an Elekta XVI system of Versa HD linear accelerator, and these images included about 1,200 projections covering an arc of 200°, and each projection had an exposure level of 0.32 mAs. The 10 phases of 4D-CBCT images were created and represented as from T00 to T90, from which T00 and T50 are the peak-inhalation phase and the peak-exhalation phase. Averaged CBCT (AVG-CBCT) was obtained from 10 phases of data. The dimension of the 4D-CBCT images with a pixel size of 2 mm × 2 mm is 205 × 205 on the axial plane. The slice thickness of the 4D-CBCT image is 2 mm. The SPECT-VIs were acquired on a Discovery NM 670 SPECT/CT scanner. Before the scan, each patient, in the supine position, inhaled 99mTc-Technegas, an ultra-fine suspension of carbon nanoparticles labeled with technetium, to assure 30 MBq of activity within the lungs, followed by SPECT acquisition performed using 3° steps through a 360° acquisition at 20 s per view. A co-registered low-dose CT image was acquired for attenuation correction and anatomical reference, and the SPECT-VI was reconstructed with a dimension of 64 × 64 × 64 and a pixel size of 8.8 mm × 8.8 mm × 8.8 mm. The 4D-CBCT and SPECT-VIs were acquired with time intervals of 7 days (median).

### Image Preprocessing

The SPECT ventilation images and 4D-CBCT images were registered using a two-step scheme. Firstly, the low-dose CT image, simultaneously acquired with the SPECT ventilation image, was rigidly registered with AVG-CBCT using MIMvista 6.8 (MIM Software Inc., Cleveland, OH, USA). Then, the registration shift was applied to align the SPECT ventilation image and AVG-CBCT. The aligned SPECT ventilation image was interpolated to maintain the spatial resolution consistent with 4D-CBCT images, and then all images were cropped to a dimension of 192 × 192 in the axial plane.

Using an intensity-based segmentation algorithm in MIMvista, the lung parenchyma was segmented on each phase of 4D-CBCT images with CT Hounsfield units in the range [−999, −250]. The main-stem bronchi and trachea were removed from each phase of the lung parenchyma. The binary lung masks of each phase of 4D-CBCT were generated and applied to define the spatial region for lung function quantification and following analysis. For patients whose imaging field-of-view (FOV) does not cover the lungs completely, we only analyzed the lungs within the FOV.

### Computation of Deep Learning-Based CBCT-VI

**Figure 1:**
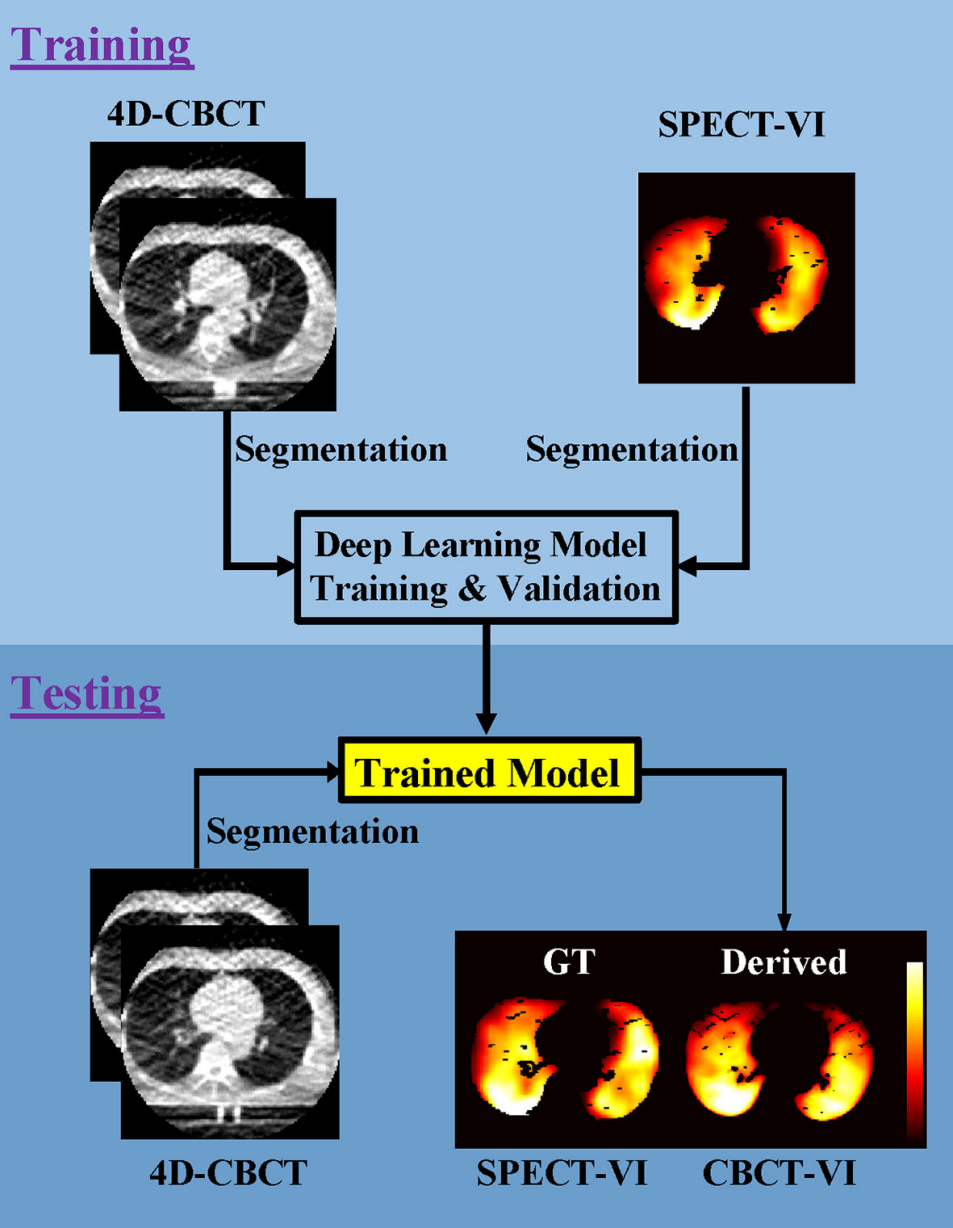
Workflow for the training and testing pipelines for deriving lung 4D cone-beam computed tomography ventilation images (CBCT-VI) and comparing it to the ground truth of a clinical gold-standard lung SPECT ventilation image (SPECT-VI).

The workflow of training and testing a deep learning model for deriving the lung CBCT-VI is shown in **Figure 1**.

The training processes included (a) segmentation of 4D-CBCT and SPECT images in lung binary masks; (b) the 10 phases of 4D-CBCT (T00-T90) and the two phases of 4D-CBCT (T00 and T50) with sizes of 192 × 192 × 10 and 192 × 192 × 2 as model input, respectively, and the SPECT-VI with size of 192 × 192 × 1 as model label; (c) the models trained on single slices; and (d) two models for deriving the CBCT-VIs [CBCT-VI_DL(1)_ for 10 phases and CBCT-VI_DL(2)_ for two phases] obtained.

The testing processes included (a) segmentation of a new patient 4D-CBCT image in lung binary masks, (b) the new patient CBCT-VI derived from the trained model, and (c) the SPECT-VI and CBCT-VI compared to evaluate the performance of the trained model.

**Figure 2:**
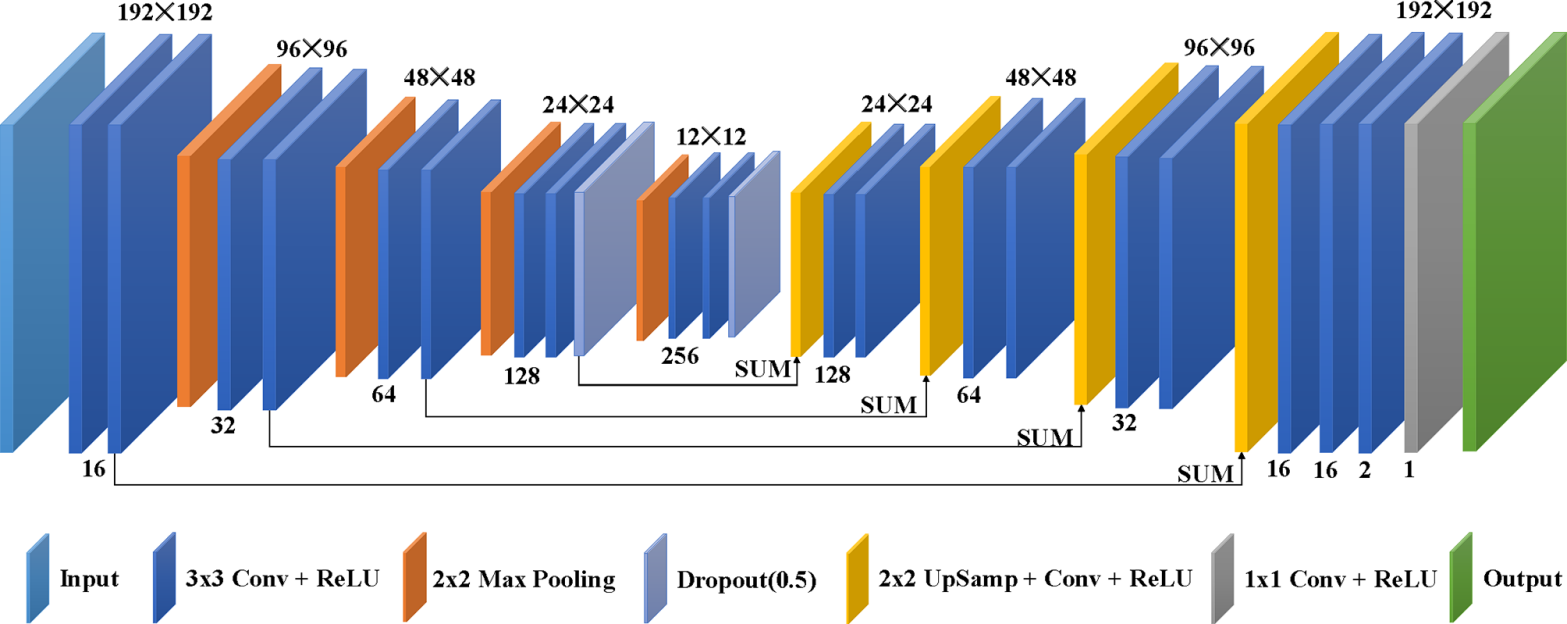
Architecture of the deep learning model for deriving CBCT-VI.

The deep learning model used in this study, as shown in **Figure 2**, is from the modified U-net (14), which was revised to use different phases of clinical 4D-CBCT images as network input and yield lung ventilation images. The architecture of the deep learning model consists of a contractive path and an expansive path. The contractive path to achieve down-sampling applies duplicated two 3 × 3 convolutions, each coming after a rectified linear unit (ReLU) and 2 × 2 max pooling with stride 2. The dropout technique was used to avoid over-fitting during the down-sampling. The expansive path of every step to achieve up-sampling consists of 2 × 2 up-sampling added with one 3 × 3 convolution and ReLU, a concatenation with the correspondingly contractive path, and two 3 × 3 convolutions, each coming after a ReLU. The architecture concludes with one 3 × 3 convolution and one 1 × 1 convolution, and each comes after a ReLU.

The training and testing processes included 1,338 slice samples in 28 patients. A sevenfold cross-validation process was applied to train and test the model. The 28 patients were randomly separated into seven equal parts, each of which was called a fold. The model was trained (80% data) and validated (20% data) on six folds of the data samples at a time and then tested on the remaining fold data samples. This process was repeated seven times so that each fold of the data has a chance to become the test dataset.

The model was trained from scratch, and the filter weights for rectifiers were initialized using “he_normal” method, allowing for extremely deep models to converge (15). The RMSprop algorithm (16) was adopted to minimize the loss function of mean squared error with a mini-batch size of 4. The loss function values in the validation datasets were monitored during training process. The initial learning rate was set to 0.0001 and automatically reduced with a drop rate of 0.2 if the validation loss value did not decrease after 10 epochs. The early-stopping technique (the training process stopped when the validation loss value did not decrease after 15 epochs) and data augmentation (flip, rotate, scale, or shift training data) were used to avoid over-fitting. The minimum loss function value in validation datasets corresponding to the well-trained model was kept for testing new patients. The model was implemented in Keras with Tensorflow as the backend. One NVIDIA 2080Ti GPU graphics card with 11 GB memory was used in this study, and it takes approximately a few seconds to obtain CBCT-VI for a new patient.

### Computation of DIR-Based CBCT-VI

For computation of the DIR-based CBCT-VI, the density-change- and Jacobian-based (HU and JAC) methods were applied to compute the CBCT-VI_HU_ and CBCT-VI_JAC_. The computation process of DIR-based CBCT-VI was described as follows: the peak-inhalation T00 and peak-exhalation T50 images of 4D-CBCT were deformed registration using a constrained intensity-based, free-form DIR algorithm (17) from the MIMvista 6.8 software, and then the deformable vector fields (DVFs) between T00 and T50 images were obtained for computing the DIR-based CBCT-VI_HU_ and CBCT-VI_JAC_.

For the computation of CBCT-VI_HU_, the DVFs can project the unit of inhale lung CBCT voxels into the exhale image field. The density change-based CBCT-VI_HU_ can be calculated using the method proposed by Castillo et al*. (* 8):


(1) $$\text{CBCT}-\text{V}{\text{I}}_{\text{HU}}=1000\frac{\left({\tilde{H}}_{T00}^{VOI}-HU_{T50}\right)}{HU_{T50}(1000+{\tilde{H}}_{T00}^{VOI})}$$

Where ${\tilde{H}}_{T00}^{VOI}$ was the average of the set of inhale lung CBCT voxels. Equation (1) was used for calculating each peak-exhalation T50 voxels in the lung CBCT binary masks. Finally, the density-change-based CBCT-VI_HU_ can be gained.

For the computation of CBCT-VI_JAC_, the changes of local lung volume were computed using the DVFs of Jacobian transformation that projects peak-inhalation T00 to peak-exhalation T50. The Jacobian-based CBCT-VI_JAC_ was computed using the formula from Reinhardt’s study (12):


(2) $$\text{CBCT}-\text{V}{\text{I}}_{\text{JAC}}=\text{J}-1,$$

where *J* was the Jacobian determinant of the displacement vector u and computed using the following formula:


(3) $$J=\left|\begin{array}{ccc} 1+\frac{\partial u_{x}}{\partial x} & \frac{\partial u_{x}}{\partial y} & \frac{\partial u_{x}}{\partial z}\\ \frac{\partial u_{y}}{\partial x} & 1+\frac{\partial uy}{\partial y} & \frac{\partial uy}{\partial z}\\ \frac{\partial u_{z}}{\partial x} & \frac{\partial u_{z}}{\partial y} & 1+\frac{\partial u_{z}}{\partial z} \end{array}\right|$$

Finally, the Jacobian-based CBCT-VI_JAC_ was obtained.

### Post-Processing of CBCT-VIs

For each patient, the CBCT-VIs obtained from those methods were firstly multiplied with peak-exhalation T50 lung binary masks, and then these CBCT-VIs were normalized by 90th percentile ventilation values. To reduce the influence of image noise, the CBCT-VIs finally went through a smoothing with a 9 × 9 × 9 box median filter.

### Spearman’s Correlation and Dice Similarity Between CBCT-VIs and SPECT-VI

In voxel-wise correlation comparisons, Spearman’s correlation *r*_s_ was calculated through the whole lung between each of CBCT-VIs and SPECT-VI for each subject. The value *r*_s_ represents the degree of correlation between two distributions, and the correlation *r*_s_ is in the range of [-1, 1], with -1 indicating a perfect negative correlation and +1 indicating a perfect positive correlation.

In similarity comparisons, the CBCT-VIs and SPECT-VI were equally divided into high, medium, and low functional lung (HFL, MFL, and LFL) regions using one-third of the highest and lowest ventilation values for each patient. The similarity of different functional lung regions between SPECT-VI and each CBCT-VI was evaluated using a dice similarity coefficient (DSC) for each patient:


(4) $$\text{DSC}(A,B)=\frac{2\left|A\cap B\right|}{\left|A\right|+\left|B\right|},$$

where *A* indicates functional lung volumes in SPECT-VI, and *B* indicates the same functional lung volumes in CBCT-VI_DL(1)_, CBCT-VI_DL(2)_, CBCT-VI_HU_, or CBCT-VI_JAC_.

An averaged DSC for a different functional lung was computed to evaluate the overall similarity.


(5) $$\text{DS}{\text{C}}^{\text{avg}}=\frac{1}{3}\sum_{i=1}^{3}{\text{DSC}}_{i}$$

where *i* indicates different functional lung. One-factor ANONA model was applied for the statistical analysis of the averaged DSC for different methods using Tukey’s honestly significant difference procedure, where this model is a four-level factor consisting of $\text{DS}{\text{C}}_{\text{DL}(1)}^{\text{avg}}$ , $\text{DS}{\text{C}}_{\text{DL}(2)}^{\text{avg}}$ , $\text{DS}{\text{C}}_{\text{HU}}^{\text{avg}}$ , and $\text{DS}{\text{C}}_{\text{JAC}}^{\text{avg}}$ .

## Results

**Figure 3:**
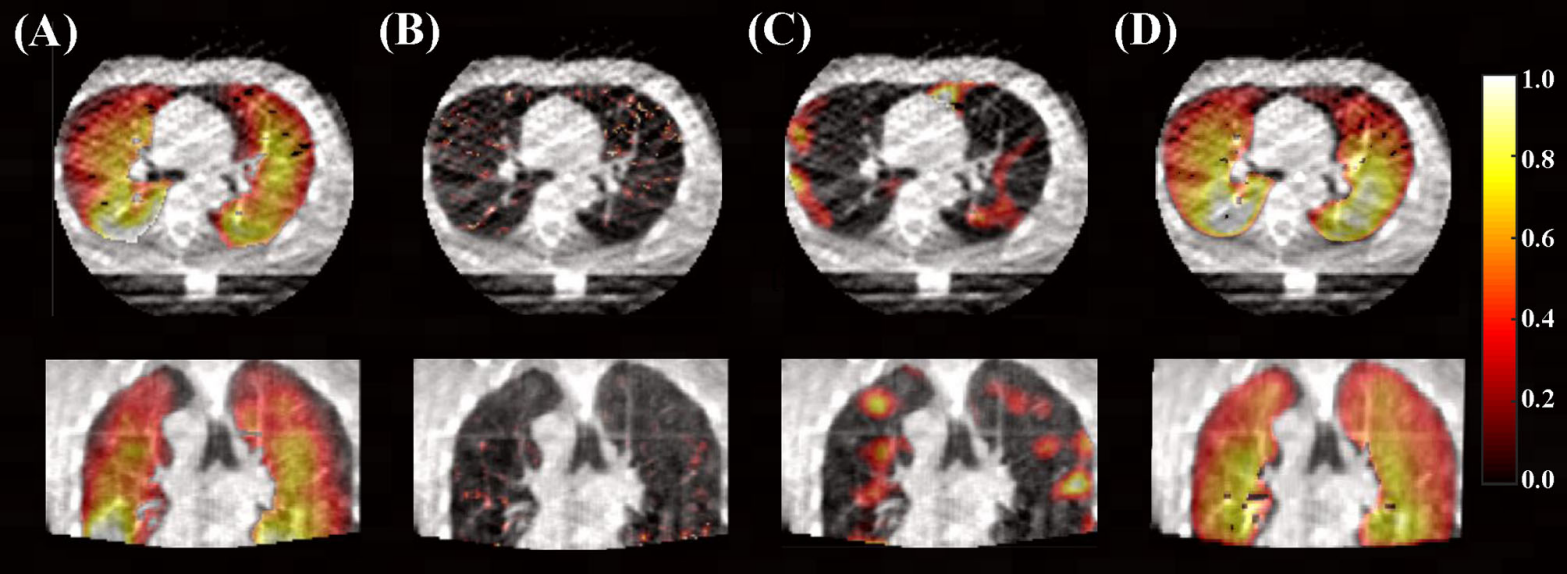
Lung ventilation images are superimposed on the peak-exhalation phase of 4D-CBCT in axial and coronal planes. **(A)** Clinical gold-standard SPECT ventilation image and **(B)** ventilation image derived from density-change-based method, **(C)** Jacobian-based method, and **(D)** deep learning-based method (10 phases as input).

For qualitative results, **Figure 3** demonstrates the distributions of derived ventilation images from different methods against the clinical gold-standard SPECT ventilation image for a specific patient. The lung ventilation images generated from different methods look very different, among which the lung ventilation image produced by the deep learning method looks closer to the clinical gold-standard SPECT ventilation image when compared with images generated from density-change-based and Jacobian-based methods.

**Figure 4:**
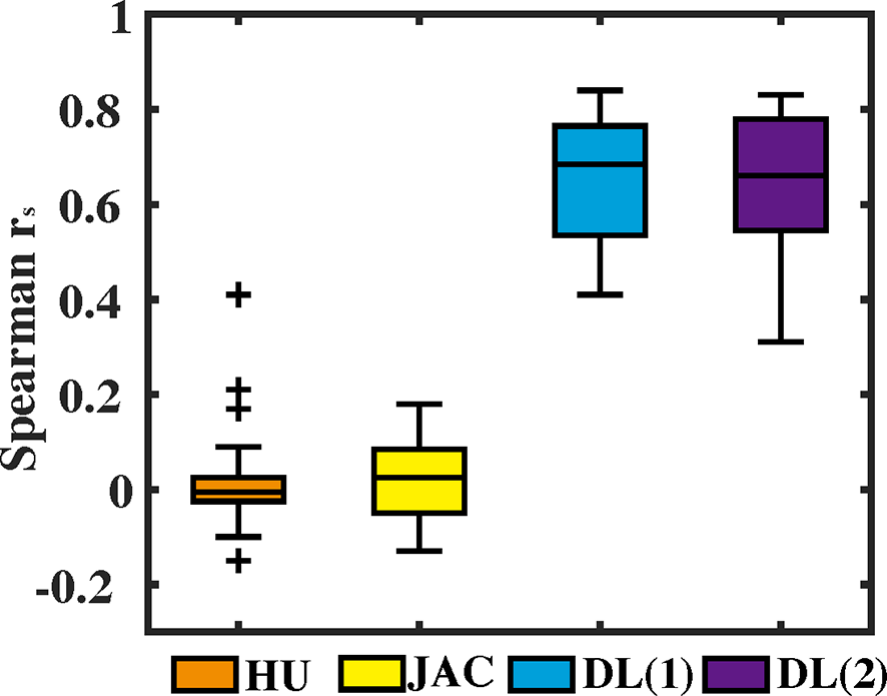
The voxel-wise Spearman correlation *r*_s_ values between the 4D cone-beam computed tomography ventilation image and the clinical gold-standard SPECT ventilation image are presented in box plot format for different methods (density-change-based method, Jacobian-based method, and deep learning-based method).

For voxel-wise quantitative results, Spearman’s correlation *r*_s_ values between CBCT-VI and SPECT-VI for the different methods are presented in **Figure 4** for all the subjects. The correlation values (mean ± SD) for HU and JAC methods were 0.02 ± 0.10 and 0.02 ± 0.10, respectively. This showed a weak correlation. The correlation values for the deep learning method were 0.65 ± 0.13 and 0.65 ± 0.15 for different input data formats. The correlation was greatly improved in comparison with HU and JAC methods, and the different input data format had no effect on the correlation results.

For similarity quantitative results, the sevenfold cross-validation DSC values between CBCT-VI and SPECT-VI of different functional lung regions for the deep learning method are summarized in **Table 1** for all the subjects. For 10 phases of 4D-CBCT as model input, the averaged DSC values of HFL, MFL, and LFL were 0.60 ± 0.08, 0.47 ± 0.05, and 0.70 ± 0.06 across the seven folds. For peak-inhalation and peak-exhalation of 4D-CBCT as model input, the averaged DSC values of HFL, MFL, and LFL were 0.59 ± 0.08, 0.46 ± 0.07, and 0.70 ± 0.08 across the seven folds. These two averaged DSC values have no significant statistical difference.

**Table 1:** The sevenfold cross-validation dice similarity coefficient (DSC) results between CBCT-VI_DL_ [CBCT-VI_DL(1)_ and CBCT-VI_DL(2)_] and SPECT-VI of high functional lung (HFL), medium functional lung (MFL), and low functional lung (LFL) regions and their average values are summarized.

Fold number	DSC [CBCT-VI_DL(1)_]				DSC [CBCT-VI_DL(2)_]			
	HFL	MFL	LFL	AVG	HFL	MFL	LFL	AVG
Fold 1	0.62 ± 0.05	0.46 ± 0.02	0.69 ± 0.05	0.59 ± 0.03	0.69 ± 0.03	0.52 ± 0.04	0.75 ± 0.04	0.65 ± 0.04
Fold 2	0.56 ± 0.10	0.43 ± 0.05	0.68 ± 0.04	0.55 ± 0.06	0.50 ± 0.06	0.39 ± 0.04	0.66 ± 0.04	0.52 ± 0.04
Fold 3	0.64 ± 0.11	0.50 ± 0.07	0.72 ± 0.07	0.61 ± 0.08	0.65 ± 0.10	0.51 ± 0.09	0.74 ± 0.09	0.63 ± 0.09
Fold 4	0.52 ± 0.12	0.41 ± 0.09	0.67 ± 0.10	0.53 ± 0.10	0.57 ± 0.12	0.46 ± 0.08	0.70 ± 0.14	0.58 ± 0.11
Fold 5	0.70 ± 0.05	0.55 ± 0.05	0.77 ± 0.04	0.67 ± 0.05	0.57 ± 0.11	0.43 ± 0.09	0.69 ± 0.07	0.56 ± 0.09
Fold 6	0.49 ± 0.06	0.42 ± 0.03	0.66 ± 0.04	0.52 ± 0.03	0.50 ± 0.08	0.42 ± 0.05	0.63 ± 0.05	0.52 ± 0.06
Fold 7	0.66 ± 0.08	0.51 ± 0.04	0.75 ± 0.05	0.64 ± 0.05	0.66 ± 0.07	0.52 ± 0.07	0.73 ± 0.11	0.64 ± 0.08

**Figure 5:**
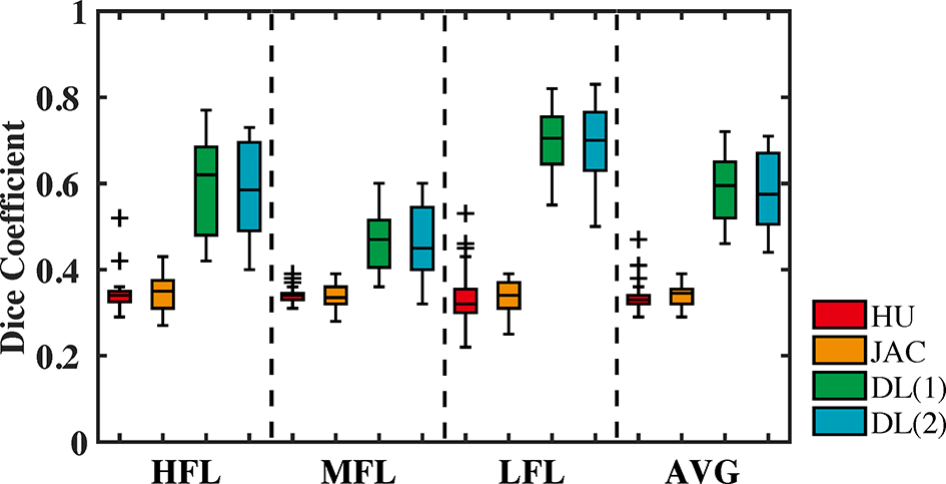
The dice similarity coefficient values between CBCT-VI and SPECT-VI of different functional lung (high, medium, and low and their average) regions for different methods (density-change-based method, Jacobian-based method, and deep learning-based method) are displayed in the form of a box plot.

Furthermore, the similarity quantitative results of different functional lung regions between CBCT-VI and SPECT-VI from the different methods are presented in **Figure 5** for all the subjects, and the DSC values of different functional lung regions are shown in **Table 2**. The averaged DSC were 0.34 ± 0.04, 0.34 ± 0.03, 0.59 ± 0.08, and 0.58 ± 0.09 for CBCT-VI_HU_, CBCT-VI_JAC_, CBCT-VI_DL(1)_ and CBCT-VI_DL(2)_ methods, respectively.

**Table 2:** The dice similarity coefficient (DSC) values between 4D cone-beam computed tomography (CBCT) ventilation images from different methods and clinical standard SPECT ventilation image for different functional lung regions and the statistical differences for these different methods.

	DSC				*P*-value			
	High functional lung (HFL) region	Medium functional lung (MFL) region	Low functional lung (LFL) region	Average	HFL	MFL	LFL	Average
CBCT-VI derived methods								
HU	0.34 ± 0.04	0.34 ± 0.02	0.34 ± 0.06	0.34 ± 0.04				
JAC	0.34 ± 0.04	0.34 ± 0.03	0.34 ± 0.04	0.34 ± 0.03				
DL (1)	0.60 ± 0.10	0.47 ± 0.07	0.70 ± 0.07	0.59 ± 0.08				
DL (2)	0.59 ± 0.10	0.46 ± 0.08	0.70 ± 0.08	0.58 ± 0.09				
Comparison for the different methods								
HU and DL (1)/HU and DL (2)					<10^−8^/10^−8^	<10^−8^/10^−8^	<10^−8^/10^−8^	<10^−8^/10^−8^
JAC and DL (1)/JAC and DL (2)					<10^−8^/10^−8^	<10^−8^/10^−8^	<10^−8^/10^−8^	<10^−8^/10^−8^
HU and JAC					0.9999	0.9868	1.0000	1.0000
DL (1) and DL (2)					0.9964	0.9916	0.9971	0.9953

We found in the DSC values that there were significant differences between the DIR-based algorithm and the deep learning-based algorithm in deriving CBCT ventilation images. The deep learning-based method for generating CBCT ventilation images has the greatest similarity to the clinical-standard SPECT ventilation image compared to HU and JAC methods with *p*-values <10^−8^ (as shown in **Table 2**), and there was no significant difference for the two forms of data as deep learning model input.

## Discussion

It is necessary to monitor the lung function changes of thoracic cancer patients during radiotherapy. 4D-CT imaging is usually used for simulation positioning in clinical practice before radiotherapy and not used as routine position verification during radiotherapy, while 4D-CBCT imaging as patient position verification during treatment is already available for many institutions. Ventilation images derived from 4D-CBCT can monitor the lung function changes during radiotherapy.

However, the existing algorithms on computing ventilation images are highly dependent on the DIR (8, 12, 13). Inaccuracies from the DIR algorithms would affect the accuracy of the derived ventilation images. Compared to 4D-CT, the reduced image quality of 4D-CBCT would further impact the precision of the DIR. It is difficult to use the existing algorithms to obtain accurate results of ventilation images from 4D-CBCT. As far as we know, the current study is the first to apply the DIR-independent deep learning method to derive ventilation images from 4D-CBCT. The generation process of CBCT-VI_DL_ did not involve any calculation of DIR. Unlike DIR-dependent methods, the deep learning method avoids any inaccuracies caused by DIR algorithms. Furthermore, previous studies have mainly investigated the correlations between 4D-CBCT and 4D-CT scan-based ventilation images (10, 11), unlike the previous studies which focused on the correlation between 4D-CT and 4D-CBCT ventilation images. In this study, we investigated the clinical gold-standard SPECT ventilation image as the output data to train the deep learning model, and this is also, for the first time, to validate a CBCT ventilation image with a SPECT ventilation image. The current study also makes an extensive comparison with the DIR-based HU and JAC methods to investigate the accuracy of the ventilation images derived from deep learning method. A significant improvement in correlation and similarity between CBCT-VI and SPECT-VI is observed when compared with DIR-based HU and JAC methods. The voxel-wise correlation was 0.02 ± 0.10 and 0.02 ± 0.09 for HU and JAC methods, while the correlation values were greatly improved with 0.65 ± 0.13/0.65 ± 0.15 for DL method. The averaged DSC values for HU, JAC, and DL methods were 0.34 ± 0.04, 0.34 ± 0.03, and 0.59 ± 0.08/0.58 ± 0.09, respectively. The deep learning method demonstrated the highest correlation and similarity (*P*-value <10^−8^) in deriving ventilation images. We here also investigated two forms of 4D images as model input. The analytic algorithms usually used two phases of peak-inhalation and peak-exhalation of 4D images to calculate ventilation images. Except for those two phases of 4D images, 10 phases of 4D images as input are also tested. The current study found that there was no significant difference between the two different forms of deep learning model input.

In theory, if the 2.5-dimensional (2.5D) or 3D network considered more spatial information, it achieve better results. Therefore, we also investigated 2.5D network to derive CBCT-VI. The 2.5D network not only considered the adjacent spatial information but also had the advantage of having relatively larger training samples, maintaining an in-plane finer resolution, and requiring a lower GPU memory compared with 3D network. In this study, the training and testing datasets only have 28 patients but including 1,338 slice samples, so the model was trained based on single slices with or without considering the adjacent slices. Meanwhile, a sevenfold cross-validation process was applied to train and test the model to make sure of the stability of the results. For 2.5D network, taking input data of 10 phases of 4D-CBCT as an example, the dimension of input data was changed from 192 × 192 × 10 to 192 × 192 × 30, in which an adjacent one slice was added for each phase. The averaged Spearman correlation was 0.67 (0.65 for 2D network), and the similarity of averaged functional lungs was 0.60 (0.59 for 2D network) for all subjects. These results are presented as supplementary materials (**Supplementary Tables S1**, **S2**). From the preliminary experimental results, we can see that the Spearman correlation and the similarity of different functional lungs make a little improvement but have no significant difference. The 2.5D network is not obviously superior to the 2D network in the task of deriving ventilation from 4D-CBCT. A possible explanation, we think, is that the motion information from different phases, not spatial information, is the key for the task, and this has already been considered in different channels for the 2D network.

In previous studies, ventilation images derived from high-quality 4D-CBCT scans have high correlations with ventilation images derived from 4D-CT. The authors Woodruff et al. (10) and Jensen et al. (11) from different groups used two different methods to improve the image quality of 4D-CBCT to help improve the accuracy of the generated ventilation images. In the study by Woodruff et al., the 4D-CBCT images were acquired by increasing the projection images, that is, by increasing the acquisition time to improve the image quality, where a correlation of 0.64 between 4D-CT- and 4D-CBCT-based ventilation images was observed. In the study by Jensen et al., they utilized a fast 4D-CBCT image acquisition technique, but the 4D-CBCT image quality was improved using modified projection correction and iterative reconstruction algorithms, where a sample mean correlation of 0.38 between 4D-CT- and 4D-CBCT-based ventilation images was observed. The current study acquired the 4D-CBCT images without increasing the projection images and applying any algorithms to improve the image quality, and a correlation of 0.65 between 4D-CBCT- and SPECT-based ventilation images was observed. These results can reflect the ability of the deep learning model to correlate features of 4D-CBCT with SPECT ventilation image. The model architecture includes many convolutional layers used for feature extraction of 4D-CBCT images. In fact, the convolutional process would also have played a role in improving the image quality of 4D-CBCT images. These results were also consistent with our previous study (18) on deriving 4D-CT ventilation images using deep learning method, and a correlation of 0.73 between 4D-CT- and SPECT-based ventilation images was reported. The correlation improvement for lung ventilation images may mainly come from the difference in image quality between 4D-CT and 4D-CBCT. Meanwhile, the results from this and our previous study were also consistent with the work of Porter et al. (19) on synthetic pulmonary perfusion images from 4D-CT, and a Spearman correlation of 0.7 between 4D-CT- and SPECT-based perfusion images was reported. In this study, to reduce the influence of image noise, all the ventilation images finally went through a smoothing with a 9 × 9 × 9 box median filter. The filter size would affect the correlation results, and we investigated how the box filter size would impact the correlation results. We recalculated the correlations by using 7 × 7 × 7 and 11 × 11 × 11 box median filters, and the averaged correlations were 0.62 ± 0.14 and 0.68 ± 0.13, respectively. The preliminary results show that the correlations have been significantly improved with the increase of the box filter size.

Furthermore, the study by Jensen et al. found that the image acquisition time interval between 4D-CT and 4D-CBCT would be a factor affecting the accuracy of the generated ventilation images. In the study by Woodruff et al., it was mentioned that the acquisition of 4D-CT and 4D-CBCT data was on the same day, and the study by Jensen et al. mentioned that the acquisition time interval of 4D-CT and 4D-CBCT was a median of 10 days. The correlation result from the study by Woodruff et al. looks better. The time interval of our study was a median of 7 days between the acquisition of 4D-CBCT and SPECT images. Even though the time interval in this study is longer than that in the study of Woodruff et al., the correlation between 4D-CBCT- and SPECT-based ventilation images derived using deep learning method looks better than the correlation between 4D-CBCT- and 4D-CT-based ventilation images measured using a DIR-based method. Additionally, further efforts are needed to update the current network performance to further improve the accuracy of CBCT-VI to make a better preparation for clinical application in the future. The 4D-CBCT and SPECT image data are limited in the current study. It would have been more helpful to improve the accuracy and to extract dynamic lung function changes during radiotherapy if more image data had been available.

## Conclusions

In summary, this study demonstrated a deep learning method for deriving CBCT-VI from clinical 4D-CBCT data and made a validation of CBCT-VI with the clinical gold-standard SPECT-VI. The results from the current study show that the deep learning model can be a steppingstone to improve the accuracy and efficiency of the derived ventilation images from clinical 4D-CBCT data. The model can be used for generating CBCT-VI, which could be potentially used for monitoring the regional lung function (20) in the future and guiding the adjustment of following treatments to avoid irradiating the high functional lung region during treatment.

## Data Availability

The original contributions presented in the study are included in the article/**Supplementary Material**, further inquiries can be directed to the corresponding author.

## Ethics Statement

The studies involving human participants were reviewed and approved by The Clinical Research Committee and the Ethics Committee at the Cancer Hospital, Chinese Academy of Medical Sciences. The patients/participants provided their written informed consent to participate in this study.

## Author Contributions

ZL, YT and JD contributed to the conception and design of the study. JD and KM provided administrative support. ZL, YT, WW, XW, TZ, and NB provided the materials or patients of the study. ZL, YT, and JM organized the database. ZL and YT performed the data analysis and interpretation. ZL wrote the first draft of the manuscript. All authors contributed to the article and approved the submitted version.

## Funding

This work was supported by the National Natural Science Foundation of China (11905295, 81502649, and 11875320), the Beijing Hope Run Special Fund of Cancer Foundation of China (LC2021B01), and the Natural Science Foundation of Beijing Municipal (7204295).

## Conflict of Interest

The authors declare that the research was conducted in the absence of any commercial or financial relationships that could be construed as a potential conflict of interest.

The reviewer YZ declared a shared affiliation with the authors to the handling editor at the time of review.

## Publisher’s Note

All claims expressed in this article are solely those of the authors and do not necessarily represent those of their affiliated organizations, or those of the publisher, the editors and the reviewers. Any product that may be evaluated in this article, or claim that may be made by its manufacturer, is not guaranteed or endorsed by the publisher.
