# Supplementary material for: Deriving Pulmonary Ventilation Images From Clinical 4D-CBCT Using a Deep Learning-Based Model
Source: Front Oncol. 2022 May 2;12:889266. doi: 10.3389/fonc.2022.889266 (PMC9109610; doi:10.3389/fonc.2022.889266)
Supplement: Supplementary file 1 [file DataSheet_1.docx]

Supplementary Material

# Supplementary Data

In theory, the 2.5-dimensional (2.5D) or 3D network considered more spatial information, they would be having better results. In this study, we investigated 2.5-dimensional (2.5D) network to derive CBCT-VI. The 2.5D network not only considered the adjacent spatial information, but also had the advantage of having relatively larger training samples, maintaining in-plane finer resolution, requiring lower GPU memory compared with 3D network. The model was trained on single slices with considering the adjacent slices. Taking input data of ten phases of 4D-CBCT as an example, the dimension of input data was changed from 192×192×10 to 192×192×30, in which adjacent one slice was added for each phase. The averaged Spearman correlation was 0.67 and similarity of averaged functional lungs was 0.60 for all subjects as shown in Supplementary Table 1 and Table 2.

Supplementary Table 1. The seven-fold cross-validation Spearman correlation results between CBCT-VI_DL(1)_ and SPECT-VI for 2D and 2.5D network are summarized.

|  |  | fold1 | fold2 | fold3 | fold4 | fold5 | fold6 | fold7 | *AVGF |
| --- | --- | --- | --- | --- | --- | --- | --- | --- | --- |
| 2D Network | Mean | 0.67 | 0.62 | 0.68 | 0.57 | 0.78 | 0.51 | 0.74 | 0.65 |
|  | *SD | 0.07 | 0.12 | 0.15 | 0.16 | 0.08 | 0.07 | 0.11 | 0.11 |
| 2.5D Network | Mean | 0.75 | 0.66 | 0.66 | 0.59 | 0.71 | 0.64 | 0.70 | **0.67** |
|  | *SD | 0.04 | 0.15 | 0.24 | 0.27 | 0.10 | 0.03 | 0.11 | 0.13 |

Note: *SD: standard deviation, *AVGF: averaged values over seven folds.

Supplementary Table 2. The seven-fold cross-validation similarity results between CBCT-VI_DL(1)_ and SPECT-VI of HFL, MFL and LFL, and their averaged (AVG) values for 2D and 2.5D network.

| Fold NO. | DSC (2D network) | | | | DSC (2.5D network) | | | |
| --- | --- | --- | --- | --- | --- | --- | --- | --- |
|  | HFL | MFL | LFL | AVG | HFL | MFL | LFL | AVG |
| fold1 | 0.62±0.05 | 0.46±0.02 | 0.69±0.05 | 0.59±0.03 | 0.66±0.05 | 0.51±0.03 | 0.76±0.02 | 0.65±0.03 |
| fold2 | 0.56±0.10 | 0.43±0.05 | 0.68±0.04 | 0.55±0.06 | 0.60±0.11 | 0.48±0.06 | 0.69±0.08 | 0.59±0.09 |
| fold3 | 0.64±0.11 | 0.50±0.07 | 0.72±0.07 | 0.61±0.08 | 0.63±0.18 | 0.53±0.12 | 0.73±0.12 | 0.63±0.14 |
| fold4 | 0.52±0.12 | 0.41±0.09 | 0.67±0.10 | 0.53±0.10 | 0.55±0.17 | 0.45±0.11 | 0.68±0.13 | 0.56±0.13 |
| fold5 | 0.70±0.05 | 0.55±0.05 | 0.77±0.04 | 0.67±0.05 | 0.63±0.08 | 0.46±0.10 | 0.73±0.07 | 0.60±0.08 |
| fold6 | 0.49±0.06 | 0.42±0.03 | 0.66±0.04 | 0.52±0.03 | 0.56±0.02 | 0.42±0.02 | 0.70±0.04 | 0.56±0.02 |
| fold7 | 0.66±0.08 | 0.51±0.04 | 0.75±0.05 | 0.64±0.05 | 0.61±0.09 | 0.48±0.04 | 0.74±0.06 | 0.61±0.05 |
| *AVGF | 0.60±0.08 | 0.47±0.05 | 0.70±0.06 | 0.59±0.06 | 0.60±0.10 | 0.47±0.07 | 0.72±0.07 | **0.60±0.08** |

Note: *AVGF: averaged values over seven folds.
